# Anti-Biofilm Perspectives of Propolis against *Staphylococcus epidermidis* Infections

**DOI:** 10.3390/biom14070779

**Published:** 2024-06-29

**Authors:** Virginia Vadillo-Rodríguez, Irene Fernández-Babiano, Ciro Pérez-Giraldo, María Coronada Fernández-Calderón

**Affiliations:** 1 Department of Applied Physics, University of Extremadura, 06006 Badajoz, Spain; vvadillo@unex.es; 2 Department of Biomedical Science, Area of Microbiology, University of Extremadura, 06006 Badajoz, Spain; ifernanb@unex.es (I.F.-B.); giraldo@unex.es (C.P.-G.); 3 University Institute of Extremadura Sanity Research (INUBE), 06006 Badajoz, Spain; 4 Networking Biomedical Research Centre on Bioengineering, Biomaterials and Nanomedicine (CIBER-BBN), 06006 Badajoz, Spain

**Keywords:** propolis, biofilms, *Staphylococcus epidermidis*, zeta potential, hydrophobicity

## Abstract

*Staphylococcus epidermis* has emerged as the main causative agent of medical device-related infections. Their major pathogenicity factor lies in its ability to adhere to surfaces and proliferate into biofilms, which increase their resistance to antibiotics. The main objective of this study was to evaluate the use and the mechanism of action of an ethanolic extract of Spanish propolis (EESP) as a potential alternative for preventing biofilm-related infections caused by *S. epidermidis*. The chemical composition of propolis is reported and its antibacterial activity against several strains of *S. epidermidis* with different biofilm-forming capacities evaluated. The influence of sub-inhibitory concentrations (sub-MICs) of EESP on their growth, physicochemical surface properties, adherence, and biofilm formation were studied. EESP interferes with planktonic cells, homogenizing their physicochemical surface properties and introducing a significant delay in their growth. The adherence and biofilms at the EESP concentrations investigated were decreased up to 90.5% among the strains. Microscopic analysis indicated that the planktonic cells that survived the treatment were the ones that adhere and proliferate on the surfaces. The results obtained suggest that the EESP has a high potential to be used as an inhibitor of both the adhesion and biofilm formation of *S. epidermidis.*

## 1. Introduction

One of the leading problems of 21st century medicine is the emergence of antimicrobial resistance, which is considered by the WHO (World Health Organization) to be one of the greatest threats to global health, food security, and development [1]. Antibiotic resistance is accelerated by the improper and abusive use of antibiotics and by deficiencies in infection prevention and control. It is a rapidly growing global phenomenon that constitutes one of the most serious health problems, to the point that medicine could run out of therapeutic drugs capable of managing certain infections.

The most difficult-to-eradicate infections are caused by immobilized bacteria (sessile cells) that form well-structured biofilms. All microorganisms that adhere to surfaces, whether living or inert, produce biofilms that protect them from host defenses and antimicrobial treatments. This peculiar mode of growth of microorganisms results in serious clinical consequences for the patient and a very high economic cost for the health system [2]. Current treatments for biofilm-related infections also include, other than antibiotic therapy, the use of antimicrobial peptides (AMPs). These short protein molecules have been shown to successfully disrupt bacterial membranes and interfere with biofilm formation. However, the high concentrations needed to disrupt biofilms can be toxic to human cells and AMPs are often degraded by proteases in the human body, reducing their effectiveness [3]. Combination therapy, i.e., using a combination of antibiotics, AMPs, and other agents to enhance efficacy, is also sometimes effectively applied, but it requires careful consideration of synergistic effects and patient-specific factors to avoid adverse drug interactions and increased toxicity [4,5,6]. Finally, surgical removal can be a critical solution for treating infections, especially in severe cases, but it constitutes an invasive procedure that carries a risk of complications, including tissue damage and scarring, functional impairment, pain and discomfort, etc.; moreover, not all biofilms can be completely removed, which can lead to persistent infections [7].

For these reasons, it is urgent to look for new substances capable of preventing or eliminating biofilm-related infections either individually or in synergy with other compounds. Positive interactions between different drugs and natural substances have already been reported. Rosato et al. and Jardak et al., for instance, showed that the combination of essential oils from different spices typically used in cuisine with existing antibiotics was particularly promising for the treatment of infections caused by several medical-related strains [8,9]. Nature, so rich, is a vast source of medicinal substances, which must be sought, analyzed, and tried to be included in the arsenal of drugs in clinical use. Interestingly, Nature has very special allies, the bees, which can produce propolis, a resinous waxy substance that they prepare by mixing their own salivary secretions with beeswax, along with compounds from various plants and trees. The bees use it to seal the holes in their comb and embalm the corpses of invading insects to mummify them and prevent them from rotting. It also protects their colonies from diseases due to its antiseptic and antimicrobial properties [10] As a mixture of secondary metabolites from the flora surrounding the hive, propolis can therefore be an important matrix that can be exploited for many therapeutic purposes [11] The chemical composition of propolis is very complex and largely depends on the geographical origin and specific flora at the site of collection. Therefore, these compounds are directly related to the diversity of geographical location, plant sources, and bee species.

The antimicrobial activities of propolis against different bacteria [12,13,14], yeasts [15], viruses [16], and parasites [17] are well documented. One recent study, for instance, investigated the effects of Mexican propolis on Candida albicans, showing that it not only inhibited biofilm formation but also reduced the proliferation and virulence of the biofilms [18,19] and further demonstrated that propolis collected from different regions of Hungary successfully prevented the growth of planktonic cells, inhibited biofilm formation, and caused significant degradation of mature biofilms for the methicillin-resistant and sensitive Staphylococcus aureus. A recent review also noted that several of the natural substances found in propolis, among other natural substances, hold promise due to their broad-spectrum activity and ability to disrupt biofilm architecture and reduce microbial viability within these protective structures [20]. These findings are part of a growing body of research aiming to develop propolis as a novel strategy to address the challenge of biofilms in clinical settings, particularly as traditional antibiotics become less effective. Previously, our group reported a novel ethanolic extract of Spanish propolis (EESP) with high antimicrobial activity [12,13,15], and an effort was made to understand its interaction mode with Gram-positive and Gram-negative bacterial cells [21]. By measuring alterations in the physicochemical properties of the cell surface following their incubation with EESP, it was found that the initial mechanism of action of this propolis was most likely structural, producing at least localized cell wall damage and/or perturbations of planktonic cells even at sub-inhibitory concentrations [21]. Moreover, a detailed analysis of its chemical composition revealed that this propolis contains a high amount of polyphenols and particularly flavonoids, some of them shared with olive oil samples and never detected before in propolis collected in others part of the world [12]. Additionally, all these compounds, presumably providing antimicrobial activity to EESP, are also known to be responsible for numerous health benefits, including cardiovascular diseases, anti-cancer, liver, or neurological disorders [22].

In *Staphylococcus epidermidis*, as in other bacteria, biofilm formation is an important aspect from a pathogenic point of view. This form of growth is considered a virulence factor, providing the bacterium with the ability to colonize surfaces (primarily of indwelling biomaterials, e.g., catheters and prostheses) and with an inherent resistance against antibiotics and host defensive mechanisms [23]. It is also worth noting that during antibiotic treatment, the concentrations inside many tissues can become lower than the bactericidal. These sub-inhibitory concentrations are not able to kill bacteria, but they can modify physical and chemical characteristics of the cell surface and, potentially, the expression of some virulence factors. Nonetheless, there are few studies that have examined the influence of sub-inhibitory concentrations of propolis on the growth of bacterial cells, their adherence to surfaces, and/or their ability to form biofilms. Moreover, within the *Staphylococcus* group, most of these studies have focused on assessing the antibacterial activity of propolis against *Staphylococcus aureus* [24,25] and the strains of *S. epidermidis*, primarily responsible for associated-biofilm infections on biomedical devices [2], have been hardly studied.

Accordingly, the purpose of this study was to evaluate the influence of sub-MIC concentrations of EESP on the growth and physicochemical surface properties of several *S. epidermidis* strains (i.e., strong-, moderate-, and negative-biofilm producer strains). Moreover, the activity of EESP against bacterial adhesion and subsequent biofilm formation (i.e., inhibition of biomass and metabolic activity) at sub-MIC concentrations was also investigated.

## 2. Materials and Methods

### 2.1. Chemical Analysis of EESP

#### 2.1.1. Liquid Chromatography Mass Spectrometry (LC–MS)

The analysis of EESP through LC-MS was performed as previously described [5]. Briefly, EESP samples of 1 μL were introduced into an LC-DAD-MS system, an HPLC (Agilent, Santa Clara, CA, USA) equipped with a qTOF mass analyzer 6520 Accurate Mass qTOF LC/MS. A Zorbax Eclipse PlusC 18 analytical column (100 mm × 4.6 mm, 3.5 µm Agilent) was used for separation using as mobile phase a formic acid aqueous solution (0.1% eluent A) and acetonitrile (eluent B). Elution was performed at a flow rate of 0.5 mL/min using a linear gradient as follows: initial 20% B, 20–30% B in 10 min, 30–40% B in 40 min, 40–60% B in 20 min, 60–90% B in 20 min, and 90% B for 5 min and 20% B in 1 min and finally 20% B in 4 min, with a total time of 100 min. Detection was achieved at 254 and 280 nm. The identification of the compounds was carried out by comparing the mass spectra and retention time of the samples with references compounds using commercial libraries (e.g., The Polyphenol Database [http://phenol-explorer.eu] and The Human Metabolome Database [https://hmdb.ca]; URL accessed on 8 June 2024).

#### 2.1.2. Method Validation and Quantification of Marker Compounds

The LC–MS was validated for calibration curve, limit of detection (LOD), limit of quantification (LOQ), accuracy, and precision. The standard compounds (vanillic acid, trans-ferulic acid, and quercetin) were purchased from Sigma-Aldrich (St. Louis, MO, USA) and prepared by dissolving the standard in HPLC grade ethanol to make concentrations of 0.1 to 5 μg/mL. Later, 1 μL of each standard was injected into the LC–MS, under the same experimental conditions as described above. The calibration graphs were constructed by plotting the mean peak intensity against concentration. The linearity was investigated by generating the regression plots by the least squares method and determining the correlation coefficient (*R*^2^). The limit of detection (LOD) and the limit of quantification (LOQ) was obtained from the y-intercept standard deviation (*S_b_*) and the slope (*m*) of the calibration curve; thus LOD = 3 ´ *Sb/m* and LOQ = 10 ´ *Sb/m*. Recovery rates (accuracy) were calculated for each condition and standard. Precision of the assay was verified by analyzing the samples three times in three consecutive days.

#### 2.1.3. Gas Chromatography Mass Spectrometry (GC–MS)

The GC–MS analysis of EESP was performed on a Bruker Scion GC–TQ-MS equipped with a HP-5MS column (30 m × 0.25 mm; film thickness, 0.25 µm) coupled to a SCION Triple Quadrupole (TQ) mass detector. The mass detector was operated in scan mode (*m*/*z* 45–450). The GC–MS analysis was temperature programmed from 50 °C (0.3 min hold) to 285 °C (15 min hold) at 6 °C/min. Samples were injected with a Combi PAL autoinjector using a splitless injection technique (0.6 µL injection volume). Carrier gas (He) flow was set at 1.0 mL/min. The compounds were identified by contrast of the acquired spectra (acquisition range 45–450 *m*/*z*) with the NIST spectrum library.

### 2.2. Propolis Sample Preparation for Bacterial Assays

The EESP employed was provided by “*La Virgen de Extremadura*” (Artesanos Virgen de Extremadura, S.L, Badajoz, Spain). It was collected in the southwest of Spain, particularly at the location of Herrera del Duque (i.e., 39.1624° N, 5.0494° W; Badajoz, Spain) and produced by mixing the propolis gathered twice a year (in spring and autumn) to minimize as much as possible season variability. The batch used was collected during the year 2020. The EESP samples employed were prepared as previously described [12]. Briefly, the ethanolic extract was filtered as received with a 0.20 µm syringe filter (Millipore, Merck, Darmstadt, Germany) and stored at 4 °C until use. For the different assays, serial twofold dilutions of EESP and its solvent (70° ethanol) were prepared in TSB (i.e., in Trypticase Soy Broth from BBL™, BD, Becton, Dickinson and Company, Franklin Lakes, NJ, USA) to obtain final concentrations ranging from 7450 to 29.8 μg/mL, considering that the dry weight of propolis obtained per mL of ethanolic extract for this batch was 59.6 mg/mL.

### 2.3. Bacterial Strains and Culture Conditions

Two biofilm producer strains of *S. epidermidis* were tested: *S. epidermidis* ATCC 35984 (RP62A) and ATCC 35983, provided by the American Type Culture Collection (ATCC, Manassas, VA, USA). In addition, a negative biofilm-forming strain, HAM 892 (isogenic mutant of RP62A) [26] was also studied. The strains, stored at −80 °C in porous beads (Microbank, Pro-Lab Diagnostics, Richmond Hill, ON, Canada), were inoculated in blood agar plates (OXOID LTD., Basingstoke, Hampshire, UK) and incubated at 37 °C for 24 h to obtain cultures. Subsequently, cells were cultivated in Trypticase Soy Agar (TSA) or Trypticase Soy Broth (TSB) (BBL™, BD, Becton, Dickinson and Company) depending on the assays. From overnight bacterial cultures in TSB incubated at 37 °C in a Memmert heater (Model 850, Schwabach, Germany), the bacterial inocula were prepared. Bacterial suspension was adjusted in TSB to 0.5 McFarland at 492 nm wavelength using a spectrophotometer (Helios epsilon, Thermo Fisher Scientific, Waltham, MA, USA), and diluted for the different assays.

### 2.4. EESP Antibacterial Activity and Effect on Bacterial Growth Curves

The minimal inhibitory concentration (MIC) and minimal bactericidal concentration (MBC) of EESP against planktonic cells of the three *S. epidermidis* strains were previously evaluated [12] and corroborated in this study. To determine the influence of sub-inhibitory concentrations on bacterial growth curves, *S. epidermidis* strains were cultured in TSB and adjusted to 6 × 10^6^ CFU/mL. The growth curve measurements were performed in 96-well microtiter plates (Greiner Bio-One GmbH, Frickenhausen, Germany) by a broth microdilution method, according to guidelines of the Clinical and Laboratory Standards Institute, CLSI [27]. The wells contained 100 µL of the different propolis extract concentrations (from the MIC to 1/8 of the MIC) in TSB and 100 µL of bacterial suspensions. Control cultures containing only TSB or ethanol were also included. Bacteria were cultured at 37 °C and cell growth was monitored spectrophotometrically by measuring the OD 490 at different time intervals.

### 2.5. Anti-Adhesion and Anti-Biofilm Activity of EESP

The influence of sub-inhibitory concentrations (i.e., 1/2, 1/4, 1/8 of the MIC and MIC) of EESP on adhesion and biofilm formation was determined by the microdilution method as described above. This assay was performed after an incubation time of 24 h by two methods, through the quantification of the total biofilm biomass after staining with crystal violet [26] and by the measurement of intracellular ATP. This last method determines the number of viable microbial cells in the biofilms, based on the quantification of ATP, as metabolically active bacteria indicator, and has been often successfully applied by others and our group. For both methods, 96-well polystyrene flat-bottomed microtiter plates (Greiner bio-one), transparent for the staining assay and white for the bioluminescence assay, were used. Each plate was inoculated as described above for growth curve measurements. The microplates were incubated for 24 h at 37 °C. After incubation, the wells were aspirated using a suction pump (Model FTA-2i, Biosan SIA. Riga, Latvia) and carefully washed at least three times with TSB to eliminate the non-adherent bacteria.

For the assessment of total biomass by staining, the bacteria adhering (biofilms) to the bottom of the wells were heat-fixed in a Pasteur Heraeus electronic oven (C.R. Maré, S.A., Barcelona, Spain) for at least 4 h at 60 °C, and subsequently stained with crystal violet (VC, Gram-Hucker DC; Panreac, Barcelona, Spain) for 5 min. Excess dye was removed with water, and again the samples were allowed to dry. The dye bound to the adherent bacteria was resuspended in 200 μL of glacial acetic acid (GAA, Fisher Scientific, Loughborough, UK) for 10 min. Finally, the color was measured with a plate spectrophotometer (ELx800; Bio-Tek Instruments, Inc. Winooski, VT, USA) at 492 nm.

For the ATP-bioluminescence method, the adherent viable bacteria included in the biofilms were quantified using the BacTiter-Glo™ Microbial Cell Viability Assay (Promega Corporation, Madison, WI, USA) according to the manufacturer’s instructions. Briefly, BacTiter-Glo™ reagents were added directly to the tested well and allowed to act for 5 min in the dark with gentle shaking (20 rpm) using a vibrating platform shaker (Model Rotamax 120, Heidolph Instruments, Schwabach, Germany), after which light emission (luciferin–luciferase reaction) was measured in a Fluorescence Microplate Reader (FLx800; Bio-Tek Instruments, Inc., Santa Clara, CA, USA). Previous to these experiments, the supernatants were transferred to another 96-well white polystyrene flat-bottomed microtiter plate and reader to determine the viability of the planktonic bacteria following this same procedure.

A negative control (TSB without inoculum) and two positive controls with bacteria (TSB and corresponding dilution of 70° ethanol) were also included. Duplicate wells were conducted for each condition evaluated and each assay was carried out at least three times with independent cultures in order to confirm reproducibility.

### 2.6. Physicochemical Surface Properties of Bacterial Cells

For zeta potential measurements, aliquots of bacterial suspensions grown the in absence and presence of different EESP concentrations (i.e., 1/2, 1/4, 1/8 of the MIC and a selected concentration just below of MIC) were resuspended in demineralized (DI) water to a final concentration of ~10^6^ CFU/mL. Measurements were performed at room temperature on a Zetasizer Nano-ZS (Malvern Instruments, Worcestershire, UK) using Malvern disposable folded capillary cells (DTS1070), which were rinsed with ethanol and thoroughly with DI water before use. The data obtained represent the mean value ± SD of at least three independent experiments.

The cell surface hydrophobicity (CSH) of the strains *S. epidermidis* ATCC 35983 and HAM 892 was previously determined by the microbial adhesion to hydrocarbons (MATH) test [21]. The same procedure was followed to determine the CSH of *S. epidermidis* ATCC 35984 and corroborate the previously obtained data. Briefly, bacterial cells grown in the absence and presence of the different EESP concentrations mentioned just above were washed and resuspended in 100 mM KCl to an optical density (*OD_i_*) of 0.4–0.6 at 600 nm using a horizontal-light spectrophotometer (Helios epsilon, Thermo Fisher Scientific). Next, 150 μL of hexadecane (99%, Sigma) was added to each bacterial suspension (3 mL). After vortex mixing for two periods of 30 s with an interval of 5 s between periods and a static incubation of 10 min, the optical density (*OD_f_*) was measured again at 600 nm. The percentage of cells in the hexadecane fraction (CSH) was calculated as CSH (%) = [1 − (*OD_f_*/*OD_i_*)] × 100 and used as a measure of hydrophobicity. The data obtained represent the mean value ± SD of at least three independent experiments.

### 2.7. Biofilm Architecture: Fluorescence and Scanning Electron Microscopy

For microscopy assays, overnight cultures of *S. epidermidis* ATCC 35983 and ATCC 35984 (RP62A) strains were adjusted to 6 × 10^6^ CFU/mL. Bacterial suspensions were incubated with different sub-inhibitory concentrations of EESP on round cover glasses (circles, 12 mm, thickness 0.13–0.17 mm, RS France, Wissous, France) located within 24-well microtiter plates (BioLite 24 Well Multidish, Thermo Fisher Scientific). Plates were incubated at 37 °C for two different periods of time, i.e., 12 h and 24 h.

Once the biofilm formation assay was finished, cover glasses were carefully washed twice with TSB in order to eliminate non-adherent bacteria and stained with a Live/Dead Baclight L-7012 kit (Invitrogen S.A, Barcelona, Spain), which contained SYTO 9 (green fluorescent dye to stain living cells) and propidium iodide (red fluorescent dye to stain damaged cells). A total of 400 µL of this solution was added per well and the samples were incubated in the dark for 15 min at room temperature.

The observation of bacterial biofilm and potential cell wall damage was carried out with an epifluorescence microscope (Model Leitz DIAPLAN, Wild Leitz Gmbh, Wetzlar, Germany), equipped with the software NIS-Elements BR 4.10 (Nikon Instruments INC., Melville, NY, USA). At least five images at random surface positions were recorded for each sample. These experiments were repeated at least three times with independent bacterial cultures.

Likewise, the biofilm morphology at sub-inhibitory concentrations of EESP was analyzed with a scanning electron microscope (SEM) (Quanta 3D FEG, FEI Company, Hillsboro, OR, USA). Prior to the examination, the cover glasses were fixed at room temperature with 3 wt% glutaraldehyde for 12 to 15 h, gently washed with PBS for 5 min, and dehydrated in a series of solutions of increasing ethanol concentrations (30, 50, 70, 90, and 100 vol%) for 1 h each. The samples were then dried in a vacuum chamber, coated with a thin layer of gold (≤5 nm) using an EMITECH K575K (Quorum Technologies Ltd., West Sussex, UK) sputter coater and finally the image captured.

### 2.8. Statistical Analysis

The data of EESP activity on biofilm formation were processed statistically by analysis of variance (one-way ANOVA) with the Statistical Package for the Social Sciences, version 22.0 (SPSS Inc., Chicago, IL, USA). The normal distribution of the mean of data for all concentrations and strains was previously checked using the Shapiro–Wilk normality test. All data are presented as mean values ± S.D. of at least three independent experiments. Differences were considered statistically significant at *p* values ≤ 0.05.

## 3. Results and Discussion

### 3.1. Method Validation and Chemical Analysis of EESP by LC-MS and GS-MS

Previous studies demonstrated that the antimicrobial properties of propolis can differ based on the season when it is harvested [28,29]. This is because the botanical sources of propolis change throughout the year, leading to variations in its chemical composition and, consequently, in its bactericidal effectiveness. To avoid as much as possible season variability, our samples were produced by mixing propolis gathered twice a year (i.e., spring and autumn of 2020). For the same site of collection, the chemical profile of propolis may also vary from year to year depending on the environment conditions, a dominant factor affecting vegetation. It is for this reason that the chemical profile of this new batch of EESP was likewise investigated through LC-MS and GS-MS analysis.

The experimental method was first validated for calibrations curve, limit of detection (LOD), limit of quantification (LOQ), accuracy, and precision. Calibration curves were constructed for three standards: vanillic acid (a new compound identified in EESP and in olive oil), trans-ferulic acid (as a representative of an olive oil component), and quercetin (a commonly used flavonoid as standard in calibration curves) at five concentration levels and in triplicate (Table 1). It was found that the vanillic acid peak represented a concentration of 18.81 μg/mL (equivalent to 0.32 mg/g propolis dry) and the trans-ferulic acid and quercetin peaks represented 224.97 μg/mL (equiv. 3.78 mg/g) and 45.88 μg/mL (equiv. 0.77 mg/g), respectively. The coefficients of correlation for the marker compounds were found to be at or greater than 0.99. Furthermore, the LOD and LOQ for vanillic acid were determined at 0.66 and 0.066 μg/mL, respectively, while for trans-ferulic acid and quercetin the LOD and LOD were 0.54 and 0.054 μg/mL. The accuracy (recovery) of the extraction for the standards was always greater than 98.5%. Regarding precision, the coefficient of variation was always below 2%. These validation studies show that the proposed method is reliable and sensitive allowing the chemical characterization of EESP.

**Figure 1:**
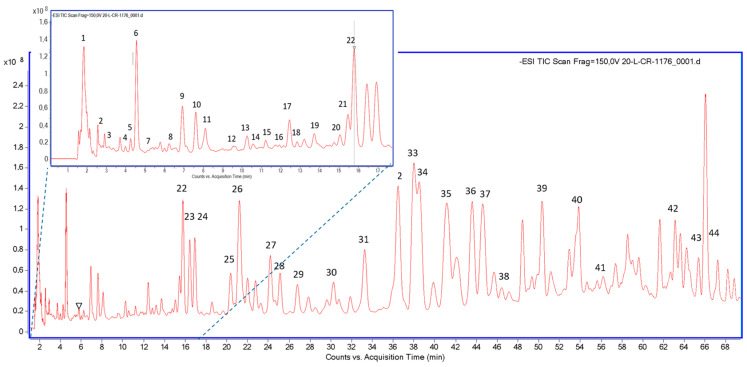
LC–MS chromatogram of EESP showing the major and minor organic peaks found in the sample.

Figure 1 shows an LC-MS chromatogram obtained for EESP, from which a total of 44 compounds were identified (vs. 56 in the previous batch) [12]; among them were flavonoids, phenolic acids, lignans, stilbene, other polyphenols (Table 2), and five volatile compounds (Table 3). Please note that the classification of the listed compounds is such that glycosides are classified by their aglycone moiety. Importantly, a total of 16 compounds were shared by both batches (highlighted in bold). Among the shared elements, 1-acetoxypinoresinol, p-HPEA-EA (p-HPEA-Elenolic acid mono-Aldehyde; ligstroside-aglycone mono-aldehyde) and vanillic acid, compounds present in extra virgin olive oil, never reported before in propolis, were detected once more [12,30,31]. Indeed, this new batch revealed a high number of phenolic compounds typically present in different olive tree tissues (like leaves, twigs or small branches, e.g., caffeic acid), and the products produced by these trees, i.e., olive oil (e.g., 1-acetoxypinoresinol) and olives (e.g., cinnamic acid), with two of them, i.e., methoxyphenylacetic acid and matairesinol, identified for the first time in a propolis sample (all marked with † in Table 2) and also found in olives and olive oil. All these compounds, together with ferulic acid, vanillic acid, hydroxycaffeic acid, and some other minor phenolic compounds (e.g., quercetin, luteolin) with well-known antimicrobial and antioxidant properties, undoubtedly add exceptional value to EESP and may be especially useful as chemical markers of this new type of propolis.

In addition, this new batch of EESP did not contain diterpenes of the typical “poplar type” patterns of Mediterranean-type propolis [32], except for pinocembrin (see Table 2), a flavonoid not found in the previous batch. Yet, it contains other diterpenes, including resveratrol 5-*O*-glucoside, matairesinol, or sesaminol, among others. It is worth noting that a recent work reported a strong correlation between the concentration of diterpenes (20-carbon terpenes) found in a large variety of propolis samples from the Greek islands of the northeast Aegean region (NEAR) and their antimicrobial activity against *S. epidermidis* [33]. Although the botanical origin of EESP remains yet unidentified, the compounds uncovered suggest olive and oak trees, and a variety of shrubs and aromatic species, in which the flora of the site of collection is very rich [34], as the possible source of EESP samples. Further studies should be conducted to confirm the botanical origin of these compounds, a necessary next step for propolis standardization purposes. Nonetheless and importantly, the chemical differences detected among this and the previous batch employed did not result in significant differences in the antimicrobial activity of the samples investigated, as evidenced in the following section.

### 3.2. Influence of Sub-Inhibitory Concentrations on Cell Growth

In line with previously published data using a different EESP batch [12], the MIC and MBC of EESP estimated in the current study for the three *S. epidermidis* strains amounted to 233 μg/mL and 466 μg/mL, respectively, i.e., this new EESP batch showed virtually the same antibacterial activity as the previous one. It is worth noting that EESP showed activity at lower concentrations than other propolis samples employed in recent studies when measuring the susceptibility of several *S. epidermidis* strains to them [33,35].

**Figure 2:**
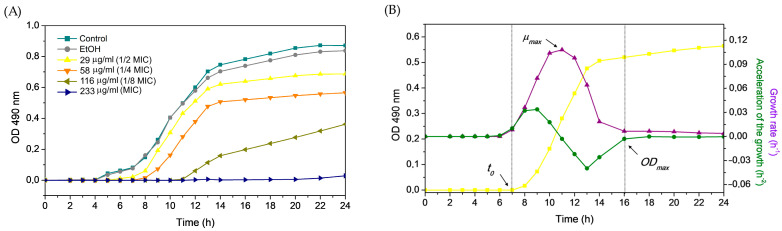
(**A**) Growth curves obtained for *S. epidermidis* ATCC 35984 under the different concentrations of EESP studied. (**B**) Example of the growth curve recorded for this strain at the EESP concentration of 58 μg/mL (¼ of the MIC) and of its first and second derivative, i.e., growth rate and acceleration of the growth, respectively, from which the main growth parameters, i.e., lag time duration (*t*_0_), maximum growth rate (*μ_max_*) and final optical density (*OD_max_*), were obtained.

**Figure 3:**
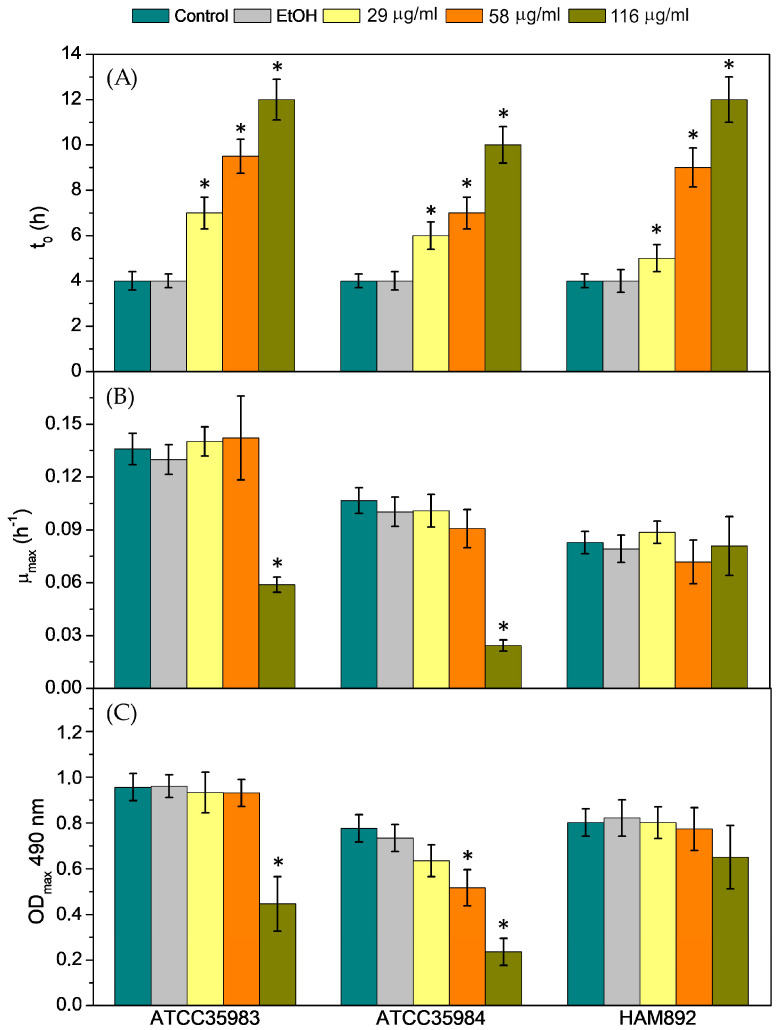
(**A**) Lag phase duration (*t*_0_), (**B**) maximum growth rate (*µ_max_*) and (**C**) final optical density (*OD_max_*) obtained from the analyses of the growth curves of the cells for the controls and the different sub-MIC concentrations of EESP investigated. Data shown as mean ± SD. * Statistically significant differences versus the control.

Accordingly, the selected EESP sub-inhibitory concentrations employed in this work were of about 116 (½ MIC), 58 (¼ MIC) and 29 μg/mL (1/8 MIC) of the MIC. As a representative image, Figure 2a illustrates the growth of *S. epidermidis* ATCC 35984 under the different sub-inhibitory concentrations of EESP investigated. For all the strains and conditions studied, these curves were decomposed into growth rate and acceleration curves (which correspond with the first and second derivatives of the growth curves, respectively) according to a novel procedure recently published [34] to determine the following growth parameters: lag time (*t*_0_), obtained from the second derivative and determined by the onset of the accelerated growth regime; maximum growth rate (*μ_max_*), given by the peak shown by the first derivative, which corresponds to the inflection point of the growth during the exponential phase; and final optical density values (*OD_max_*), marked by the point at which the deceleration phase ends, i.e., when to total number of cells tend to asymptote (Figure 2b). The results obtained are shown in Figure 3.

Figure 3a shows that all propolis sub-MIC concentrations produced a significant delay on the growth of all three strains (also observed in the example displayed in Figure 2A), as demonstrated by the observed extended lag times. In particular, their growth was progressively delayed up to ~6 to 8 h in the presence of EESP with respect to the untreated cells. EESP has been recently shown to cause localized cell wall damage at sub-inhibitory concentrations, significantly interfering with the physical integrity of the bacterial cell walls [21]. Based on these previous investigations and other works [24,36], it is suggested that the extended lag times observed here could be attributed to the bactericidal effect of propolis on a large number of cells. As a result, the surviving cells would spend more time in reaching a concentration where the OD value significantly increases above the lag level, yielding the longer lag times detected.

Remarkably, despite the delay onset of growth, cells exposed to EESP sub-MICs were found to grow exponentially at a rate comparable to that of the untreated cells, except for the biofilm producers (strong and moderate, respectively) *S. epidermidis* ATCC 35984 and ATCC 35983 at the highest sub-MIC concentration evaluated, i.e., 116.4 μg/mL (½ MIC) (Figure 3B). This parameter, i.e., the maximum growth rate (*μ_max_*), is typically considered as a measure of the growth capacity of the cells and, hence, of their physiological rigor. These later results illustrate that EESP at sub-MIC concentrations does not generally hamper the growth of the probably physiologically intact remaining cells after the destruction of many of them during the lag phase. The growth of these surviving cells is probably resumed as usual after the eradication and/or inactivation of some of the main active antimicrobial compounds of EESP. The maximum *OD_max_* values observed were also partially unaffected by the sub-MIC concentrations employed (Figure 3C), except for the strong-biofilm producer *S. epidermidis* ATCC 35984, for which a decreasing trend with the increase of antimicrobial concentration was perceived, i.e., the amount of optical density (including adherent cells, planktonic cells, and/or other excreted components) was reduced in a dose-dependent manner with respect to the control. Nevertheless, statistically significant differences with respect to the control values were only detected for this strain at the two highest sub-MIC concentrations evaluated, i.e., 58 and 116 μg/mL (¼ and ½ the MIC), and for the moderate-biofilm producer *S. epidermidis* ATCC 35983 at 116 μg/mL of EESP.

Taken together, these results revealed that the lag time (*t*_0_), rather than the maximum growth rate (*μ_max_*) or the final OD value (*OD_max_*), might be a more meaningful indicator of dose-dependent inhibitory effects on microbial growth. This is especially true for the biofilm-negative *S. epidermidis* HAM 892 strain, for which neither *μ_max_* or *OD_max_* correlated with the antimicrobial concentration. The correlations found at the highest sub-MICs employed between these last two parameters and the biofilm-former strains are likely due to the specific interaction of propolis compounds with the polysaccharide-rich most outer surface of these strains, which greatly differ from that of HAM892, non-producer of EPS. Therefore, the results presented suggest that knowledge of the extended lag times rather than *μ_max_* or *OD_max_* could potentially be of much higher applicability in properly establishing, for example, dosing regimens for some antimicrobials.

Finally, it is worth mentioning that the results obtained were not influenced by the presence of ethanol in the EESP solutions, as can be seen in the Figure 2 and Figure 3.

### 3.3. Cell Surface Physicochemical Properties

The physicochemical surface properties of the cells are most likely to experience alterations as the propolis compounds interact with the cell surface. In line with this, the cell surface charge and hydrophobicity were evaluated following the incubation of the cells with the different sub-inhibitory concentrations of EESP investigated.

**Figure 4:**
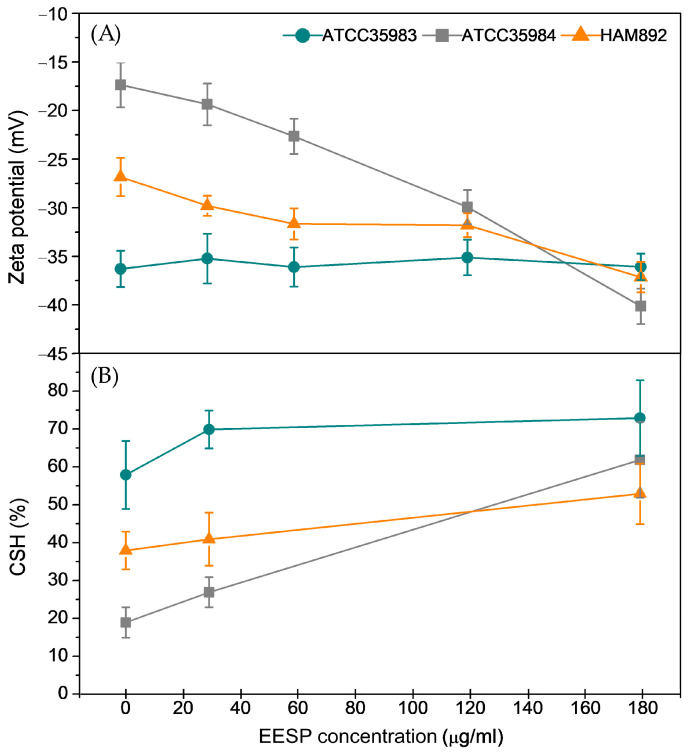
Zeta potential values (**A**) and degree of cell surface hydrophobicity assessed by MATH (**B**) measured for the strains investigated in the absence and presence of the different sub-MIC concentrations of EESP tested.

Figure 4A shows the experimentally determined zeta potentials of the three strains as a function of the concentration of EESP. As expected, untreated cells are all negatively charged, with the strong-biofilm producer *S. epidermidis* ATCC 35984 presenting the lower density of negatively charged groups. At this point it is important to emphasize that this later strain produces, among them, the higher level of the polysaccharide intercellular adhesin (PIA), a cationic (i.e., positively charged) partially deacetylated homopolymer of N-acetylglucosamine [37]. This cationic polymer is expected to neutralize partially the naturally occurring negatively charged groups on cell surfaces due to the presence of ionized carboxyl, phosphate, and amino groups [38]. Figure 4A also illustrates that, except for the moderate-biofilm producer *S. epidermidis* ATCC 35983, whose zeta potential remained unaffected, the cell zeta potentials become more negative on increasing the antimicrobial concentration, reaching a common value for all three strains at the highest sub-MIC evaluated. Interestingly, this latter finding could be indicative of a progressive accumulation of propolis compounds in the cell surface, which would explain the homogenization of the collected data. Other studies also reported a slight increase in the negative charge of *S. epidermidis* treated with different isolated propolis extracts in a concentration-dependent manner [39].

Cell surface hydrophobicity measurements also appear to reveal changes induced by EESP on the surface of the strains studied, but less significantly. Figure 4B shows the results obtained for all the conditions investigated. As can be observed, untreated cells showed a relatively low cell surface hydrophobicity, with the strong-biofilm producer *S. epidermidis* ATCC 35984 exhibiting the lowest affinity towards hexadecane, followed by its biofilm-negative mutant HAM892 and the moderate-biofilm producer ATCC 35983. This trend correlates with the relative amount of polysaccharides and proteins carried by the surface of the cells studied. In particular, a lower amount of polysaccharides (i.e., HAM 892 vs. ATCC 35984) and a higher protein content (i.e., ATCC 35983 vs. ATCC 35984 and HAM 892) have been both reported to result in higher cell surface hydrophobicity [40]. Exposure to EESP showed signs of slightly increasing the cell surface hydrophobicity in an apparent concentration-dependent manner, but statistically significant differences were only detected for the strong-biofilm producer *S. epidermidis* ATCC 35984 and its biofilm-negative mutant HAM 892 with respect to their control values. Curiously, at the highest sub-MIC concentration investigated, the presence of EESP appeared to level off to a near common value the hydrophobicity of all three strains (no statistical differences were found). It was previously suggested that the typically amphiphilic compounds that constitute propolis might preferably interact with the cells through the formation of intermolecular hydrogen bonds, with their aromatic rings remaining as extracellular residues [21]; this structural configuration would progressively convey hydrophobicity to the surface of the cells as the number of propolis compounds associated with it gradually increases.

Therefore, the physicochemical surface properties of the cells experienced detectable alterations after their interactions with sub-inhibitory concentrations of EESP. These changes might be related to the increasing accumulation of propolis compounds on the cell surface, which would explain the homogenization of the properties evaluated among the strains.

### 3.4. Influence of Sub-Inhibitory Concentrations on Adhesion and Biofilm Formation

**Figure 5:**
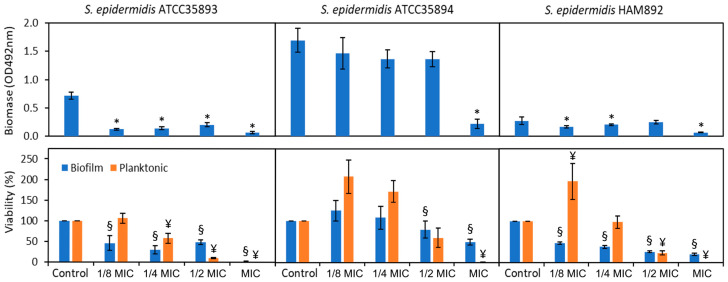
Biofilm biomass and viability of the adherent and planktonic cells determined for the three strains investigate in the absence and present of the different sub-MIC concentrations of EESP. Statistically significant differences versus the control (*, ¥, §).

Bacterial adherence to surfaces, primarily influenced by hydrophobic and electrostatic interactions, constitutes a first step for subsequent biofilm formation. Figure 5 shows the effect of the different sub-inhibitory concentrations on both the bacterial cells that remained attached to the surface and those that remained in suspension after 24 h of treatment with EESP.

For the moderate-biofilm producer strain, *S. epidermidis* ATCC 35983 (Biomass Control = 0.72 ± 0.06), all sub-MIC concentrations reduce biofilm formation significantly. Accordingly, the viability of adherent cells, i.e., the ATP signal detected, also decreases at all concentrations, with reduction percentages of 53.9, 70.0, 51.4, and 98% at the concentrations of 29, 58, 116 and 233 μ/mL (i.e., 1/8, ¼, ½ of the MIC and the MIC), respectively. As can be seen at one half of the MIC (116.4 μg/mL) the biomass (reduced by 72%) and viability of the biofilm appeared higher than expected, to the detriment of viable planktonic bacteria, which decrease considerably (90.2% reduction). Conversely, these cells were found to grow at a slower rate (*μ_max_*) and reach a lower final density (*OD_max_*) at this particular concentration. It seems that, at this concentration, the fewer cells growing and surviving succeed more easily on adhering to the surfaces (biomass) or be more metabolically active (viability). Cell surface-accumulated EESP compounds may facilitate their binding to the surface through specific interactions more than expected, as surface charge for this strain remains unchanged among the range of concentrations investigated (Figure 4A) and the observed increase of the cell surface hydrophobicity was not significant (Figure 4B).

In the highly producing strain *S. epidermidis* ATCC 35984 (biomass control = 1.69 ± 0.21), all concentrations decrease somewhat in biofilm biomass, but only at the MIC (233 μg/mL) concentration is the measured decrease statistically significant. However, biofilm viability is substantially inhibited at both one half the MIC and the MIC, i.e., at 116 and 233 μg/mL, with reduction percentages of 20.3 and 51.1%, respectively. These concentrations reduce drastically the viability of planktonic cells as well. In particular, at the EESP concentration of 233 μg/mL (MIC) there are no live planktonic cells, but the bacteria that remain attached to the surface remain highly active. For this particular strain, it is notable that although all concentrations seem to slightly reduce the attached biomass, sub-MIC concentrations of 29 and 58 μg/mL (1/8 and ¼ the MIC) increase the viability (measured as intracellular ATP) of both the adhered and planktonic cells, probably in response to the attachment of the active compounds of EESP. Nevertheless, as the dispersion of the data for this strain is wide, probably due to its high biofilm-forming capacity, no statistically significant differences with the control were detected at these lower concentrations.

Therefore, the behavior of the two biofilm producing strains against the sub-MICs of the extract studied differs: all concentrations decrease the viability within the biofilm in the moderately producing strain (*S. epidermidis* ATCC 35983), while in the highly producing strain (*S. epidermidis* ATCC 35984), some concentrations can even increase biofilm ATPase activity. These discrepancies may be associated with the nature, composition, and amount of the biofilm EPS, which differs in both strains [23]. The significant higher surface charge and hydrophobicity detected for the protein-rich EPS matrix produced by *S. epidermidis* ATCC 35983 might provide a more conductive environment for the formation of intermolecular hydrogen bonds, the recently proposed mechanism for the interaction of the amphiphilic EESP molecules with the outermost surface of the cells [21], than the polysaccharide-rich EPS matrix associated with *S. epidermidis* ATCC 35984. A high concentration of antimicrobial components of propolis in the outermost surface of the first mentioned strain presumably forces or facilitates the penetration of these compounds deeper into the cell extracellular layer, reaching even close contact with the cell wall or membrane. This would compromise, as observed, the cells biomass and viability. On the other hand, the amount of propolis components accumulated on the slightly negatively charged and hydrophilic outermost surface of the *S. epidermidis* ATCC 35984 cells at low sub-MICs is likely to be too low to promote their deeper penetration into the cell extracellular layer. In this particular case, the propolis components located on the periphery of these cells seem to help to bridge the gap between the cells and the cells and the surface and somehow activate the cells. For this strain, concentrations of one half of the MIC of EESP (116 μg/mL) and higher are needed to compromise cells biomass and viability. Curiously, this strain is the one that experiences the largest alteration in its physicochemical properties in the presence of EEPS (Figure 4), likely revealing its greater ability to adapt to the stress produced by this antimicrobial.

The behavior of the mutant strain, *S. epidermidis* HAM 892 (biomass control = 0.30 ± 0.09), appears to be intermediate between the other two strains (in agreement with its physicochemical surface properties, also found to lie in between both). All concentrations significantly decreased the viability of the adhering cells, ranging from 52.1 to 79.7%. Similar to ATCC 35983, there appears to be more biomass attached to the surface than would correspond at one half the MIC of EESP (116 μg/mL), but with a significantly reduced viability of attached and planktonic cells with respect to the control. Furthermore, as these cells do not produce the biofilm of the strain from which it originates (i.e., ATCC 35984), the observed increase in viability at the lowest EESP concentrations employed, i.e., 29 and 58 μg/mL (1/8 and ¼ the MIC), observed for its wild-type is reflected in this strain exclusively in the planktonic cells, which have probably responded with a higher ATPase activity against the attack of the EESP molecules. It is further observed that the concentration of 233 μg/mL (MIC) for *S. epidermidis* ATCC 35984 and HAM 892 is less effective than for ATCC 35983. This is noteworthy, as it corroborates that the behavior of the mutant strain is similar to that of the biofilm-producing strain from which it originates.

At this point, it should also be noted that the reductions observed in adhesion and biofilm formation are exclusively due to the action of the propolis and not to the ethanol present in the extracts, which sometimes facilitated adhesion (data available upon request). A few recent studies explored the antimicrobial properties of several of the phenolic compounds detected in EEPS, including hydroxycaffeic acid, vanillic acid, ferulic acid, and 1-acetoxypinorsinol, among others [41,42,43,44]. It was shown that all these compounds, in the isolated from, possessed inherent antibacterial properties by inhibiting bacterial adhesion and growth. Their exact mechanism of action is not yet fully understood, but it has been suggested that they exhibit these properties by affecting the bacterial cell wall and interfering with quorum sensing, a critical process in biofilm formation and maintenance.

Finally, it should be mentioned that the assay here described, was performed by the broth microdilution method (a different method than that employed for the MIC determinations due to the color of EESP, which would interfere with the values measured by spectrophotometry), and shows some bacterial viability of the cells at the MIC concentration, i.e., at 233 μg/mL.

### 3.5. Effect of Propolis Extract on the Architecture of the Biofilm

**Figure 6:**
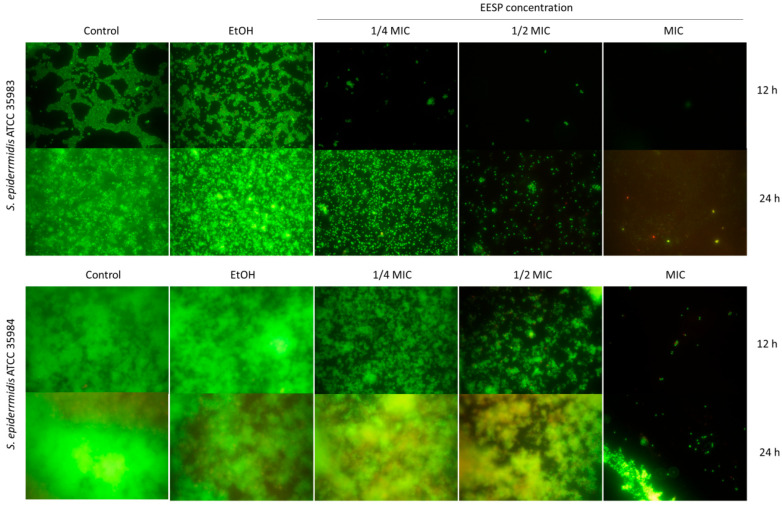
Fluorescence microscopy images of the biofilms produced by *S. epidermidis* ATCC 35983 and ATCC 35984 under the different conditions investigated (i.e., controls and sub-MIC concentrations of EESP) after an incubation time of 12 and 24 h.

Figure 6 shows fluorescence microscopy images of the biofilms produced by *S. epidermidis* ATCC 35983 and ATCC 35984 after treatment with the sub-MIC concentrations of EESP studied at different times, after 12 h (i.e., the maximum lag time detected in Figure 3), and 24 h.

After an incubation time of 12 h, it is observed that all sub-MIC concentrations substantially prevented the adherence of the cells from *S. epidermidis* ATCC 35983. This observation is maintained after an incubation time of 24 h, in full agreement with the data presented in Figure 5. *S. epidermidis* ATCC 35984, however, displays only a dose-dependent decrease in the number of live adhering cells after 12 h of contact with EESP. After 24 h, a greater bacterial proliferation, which includes the formation of bacterial aggregates and clumps, is generally observed, except at the highest EESP concentration studied, i.e., 233 μg/mL (MIC). This observation, once again, fully agrees with the data presented in Figure 5. Moreover, it is noteworthy that, regardless of the strain, all adhered cells seem to have their plasma membrane intact (i.e., they mostly emit green fluorescence), with the presence of a only a few orange-damaged cells in the biofilm produced by the strong-biofilm producer *S. epidermidis* ATCC 35984. This finding suggests that only the planktonic cells that survive undamaged during the EESP treatment are the ones that achieve the subsequent colonization of the surfaces. Finally, the images presented also prove that ethanol is not responsible for the results obtained.

**Figure 7:**
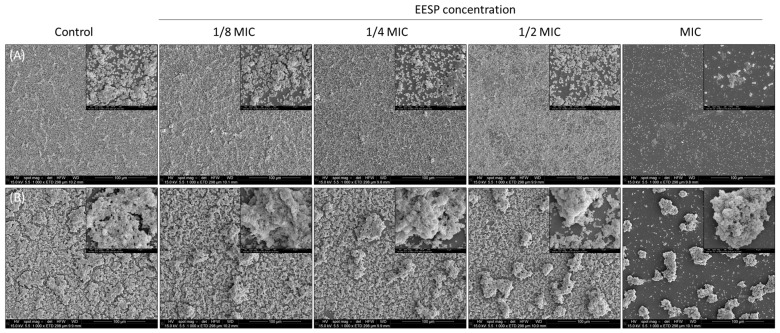
Scanning electron micrographs of the biofilms produced by (**A**) *S. epidermidis* ATCC 35983 and (**B**) ATCC 35984 under the different conditions investigated (i.e., controls and sub-MIC concentrations of EESP) after an incubation time of 24 h. Image magnification: 1000× and 10,000×.

Figure 7 shows SEM images of the biofilms produced by *S. epidermidis* ATCC 35983 and ATCC 35984 in the presence of the sub-MIC concentrations of EESP. For both strains, a dose-dependent decrease in biofilm production is generally perceived, which likely relates with the delayed onset of growth detected during the analysis of their growth curves (Figure 3A). This is most notable for the high biofilm-producing strain, for which the measured *OD_ma_*_x_ (Figure 3C) already suggested a dose-dependent decrease in the total number of cells during growth. Nonetheless, for the strain *S. epidermidis* ATCC 35983, the concentration of 116 μg/mL (½ the MIC) appeared once more (see Figure 5) to be critical, promoting cell–cell and cell–surface adhesion. Importantly, no irregularly shaped or damaged cells are detected at any of the concentrations employed. Moreover, at the MIC concentration (233 μg/mL), hardly any adhered cells are detected for *S. epidermidis* ATCC 35983 (magnitude 10,000×). For *S. epidermidis* ATCC 35984, however, it is clearly seen that the only bacteria that have managed to adhere to the surface at this concentration are those that have been able to proliferate and generate the typical mushroom-like structures of biofilms (magnitude 1000×).

Collectively, the results presented demonstrated that EESP from different batches displayed similar antimicrobial activity against several *S. epidermidis* strains despite some detected discrepancies in their chemical profile. Moreover, the sub-MIC concentrations employed were found to homogenize the cell surface physicochemical strains of the three strains evaluated, increasing their negative surface charge and hydrophobicity (Figure 4). This homogenization is remarkable, considering that the initial binding to abiotic and natural surfaces is thought to be primarily mediated by hydrophobic and electrostatic interactions [23]. As mentioned above, other groups also reported a slight increase in the negative charge of *S. epidermidis* treated with different isolated propolis extracts in a concentration-dependent manner [39] and, interestingly, this variation in charge resulted in lower adherence and higher biofilm disruption. It is suggested here that the observed homogenization of the cell surface properties likely results from the progressive accumulation of propolis compounds on the surface of the cells during the incubation time, being of special relevance for the strong-biofilm producer strain, for which the largest variations in physical–chemical properties with respect to its wild state were recorded.

Moreover, sub-MIC concentrations of EESP were found to provoke a significant and progressive delay on the growth of the strains evaluated, as demonstrated by the extended lag times detected during the analysis of the growth curves (Figure 2 and Figure 3A). This finding is attributed to the bactericidal effect of propolis on the suspended cells. Accordingly, only the surviving cells manage to adhere to the surfaces and multiply to form the characteristic biofilms of *S. epidermidis*. This would explain the dose-dependent lower adherence and biofilm development typically found for the strains investigated and the lack of adherent damaged cells revealed by microscopy analysis (Figure 6 and Figure 7).

In general terms, the *Staphylococcal* biofilm-forming capacity has been shown to be inhibited, to a larger or shorter extent, by Polish [45], Russian [46], Brazilian [47], and Portuguese propolis [35], but these and other studies also have shown that once a biofilm is formed, large amounts of propolis are required to remove some of the bacteria included in the biofilm, with a residual population always present that may contribute to recurrent infections [48]. The results here presented demonstrate that EESP has the ability to delay and partially prevent the formation of biofilms from *S. epidermidis*. This is most likely due to the decrease in the number of viable suspended cells capable of adhering to surfaces, an essential first step for the initiation and development of biofilms. This general finding suggests that EESP could potentially be used as an antibiotic adjuvant for the prevention of *S. epidermidis* infections to successfully delay the onset of biofilm formation and reduce the amount of antibiotic required during treatment. This would help, for instance, to lessen the development of antimicrobial resistance, as it has been demonstrated that propolis exhibits synergism with various antibiotics [14,24,39] or even reverses resistance to certain antibiotics [49]. Moreover, the presence of olive oil compounds in this particular propolis extract uncovers excellent biological and pharmaceutical properties that can contribute very likely to promote human health.

## 4. Conclusions

The present work demonstrated that even sub-MIC concentrations of EESP have antimicrobial activity against *Staphylococcus* spp. in vitro. In particular, the results presented suggest that these concentrations primarily interfere with suspended cells, homogenizing their physicochemical surface properties and introducing a significant delay in their growth. Only the cells that survived the treatment were found to adhere to the surfaces and proliferate. Consequently, a significant reduction of the initially adherent cells and subsequent formation of biofilms was detected for the three strains evaluated. According to these results, EESP could be considered as a potential biofilm inhibitor for *S. epidermidis*. However, despite the promising antibacterial properties of propolis, its use as an antibacterial agent has several limitations. The chemical composition of propolis can vary significantly due to differences in geographical location, plant sources, and seasonal changes, leading to considerable chemical heterogeneity that can affect its biological properties. Additionally, propolis may cause allergic reactions or toxicity at high doses. Therefore, comprehensive and in vivo validation studies are essential to ensure its safety, efficacy, and standardization. Future research should focus on identifying the specific active compounds responsible for the antibacterial properties of propolis to reduce its limitations and develop more specific and potent formulations. Moreover, combining these active compounds with conventional antibiotics might be a crucial step in overcoming antibiotic resistance, enhancing antimicrobial activity, and reducing the necessary doses of antibiotics, potentially minimizing side effects. In addition, it is worth mentioning that the presence in EESP of polyphenols also present in extra virgin olive oil, provides an exceptional added value to this extract, offering many direct benefits in terms of prevention and/or coadjuvant treatment in different pathological conditions in human health.

## Figures and Tables

**Table 1:** Equations of calibration curves for marker compounds.

Compound	Linear Range (µg/mL)	Calibration Curve	R^2^	Amount (µg/mL)
Vanillic acid	0.1–5	*y* = 36,508*x* – 689.8	0.9990	18.81
Trans-ferulic acid	0.1–5	*y* = 207,867*x* + 624.4	0.9993	224.97
Quercetin	0.1–5	*y* = 866,860*x* + 26,682	0.9993	45.88

**Table 2:** Polyphenolic compounds of the ethanolic extract of Spanish propolis (EESP) identified by LC-MS.

Compounds Identified by LC-MS						
Peak	RT (min)	Proposed Structure	Class	Formula	Mw	*m*/*z*
1	1845	**Hydroxycaffeic acid** †	PA	C_9_H_8_O_5_	196.0372	196.0377
2	2740	**Vanillic acid** †*	PA	C_8_H_8_O_4_	168.0423	168.0412
3	3138	3,4-Dihydroxyphenylacetic acid †	PA	C_8_H_8_O_4_	168.0423	168.0413
4	4067	Homovanillic acid †	PA	C_9_H_10_O_4_	182.0579	182.0568
5	4249	Sesamol	L	C_7_H_6_O_3_	138.0317	138.0306
6	4581	Caffeic acid †	PA	C_9_H_8_O_4_	180.0423	180.0419
7	5045	**p-HPEA-EA** †*	OP	C_19_H_22_O_7_	362.1366	362.1355
8	6206	Methoxyphenylacetic acid †*	PA	C_9_H_10_O_3_	166.063	166.0616
9	6916	p-Coumaric acid †	PA	C_9_H_8_O_3_	164.0473	164.0465
10	7616	**Ferulic acid** †	PA	C_10_H_10_O_4_	194.0579	194.0569
11	8097	Isoferulic acid	PA	C_10_H_10_O_4_	194.0579	194.0569
12	9540	**Cinnamic acid** †	PA	C_9_H_8_O_2_	148.0524	148.0514
13	10,253	Resveratrol 5-*O*-glucoside	S	C_20_H_22_O_8_	390.1315	390.1293
14	10,833	Quercetin 3-*O*-rhamnoside †	F	C_21_H_20_O_11_	448.1006	448.0977
15	11,215	**Apigenin 6-*C*-glucoside**	F	C_21_H_20_O_10_	432.1056	432.1032
16	11,928	Isohydroxymatairesinol	L	C_20_H_22_O_7_	374.1366	374.1337
17	12,442	3,4-dimethyl-caffeic acid	PA	C_11_H_12_O_4_	208.0736	208.0733
18	12,823	**Quercetin 3-*O*-rutinoside** †	F	C_27_H_30_O_16_	610.1534	610.1521
19	13,719	Vanillic acid-glucoside	PA	C_14_H_18_O_9_	330.0951	330.0948
20	15,046	Luteolin †	F	C_15_H_10_O_6_	286.0477	286.05
21	15,427	Quercetin	F	C_15_H_10_O_7_	302.0354	302.0331
22	15,775	**Kaempferol**	F	C_15_H_10_O_6_	286.0477	286.0467
23	16,455	**Sinapinaldehyde**	OP	C_11_H_12_O_4_	208.0736	208.0724
24	16,920	Quercetin-3-methyl ether	F	C_16_H_12_O_7_	316.0583	316.0533
25	20,419	Apigenin	F	C_16_H_14_O_4_	270.0896	270.0886
26	21,198	Pinobanksin	OP	C_15_H_12_O_5_	272.0685	272.0675
27	24,183	Kaempferol-methyl ether	F	C_16_H_12_O_6_	300.0634	300.0624
28	25,096	Kaempferol-methoxy-methyl ether	F	C_19_H_18_O_8_	374.1002	374.1019
29	28,788	Galanin-5-methyl ether	OP	C_16_H_12_O_5_	284.0685	284.079
30	30,270	**Rhamnetin**	F	C_16_H_12_O_7_	316.0583	316.0544
31	33,271	Quercetin-dimethyl ether	F	C_17_H_14_O_7_	330.074	330.0742
32	36,439	Caffeic acid isoprenyl ester	PA	C_14_H_14_O_4_	246.0982	246.0972
33	37,998	Caffeic acid isoprenyl ester (isomer)	PA	C_14_H_14_O_4_	246.0982	246.0972
34	38,529	Caffeic acid benzyl ester	PA	C_16_H_14_O_4_	270.0892	270.0845
35	41,215	Pinocembrin	F	C_15_H_12_O_4_	256.0736	256.0737
36	43,603	Caffeic acid phenyethyl ester	PA	C_17_H_16_O_4_	284.1049	284.1039
37	44,698	Pinobanksin-3-*O*-acetate	OP	C_17_H_14_O_6_	314.079	314,0781
38	47,102	Matairesinol †*	L	C_20_H_22_O_6_	358.1416	358.1389
39	50,336	Caffeic acid cinnamyl ester	PA	C_18_H_16_O_4_	296.1049	296.1149
40	53,326	**1-Acetoxypinoresinol** †*	L	C_22_H_24_O_8_	416.1471	415.1363
41	56,140	**Arbutin**	OP	C_12_H_16_O_7_	272.0896	272.0892
42	63,604	Pinobanksin-3-0-pentanoate or 2-methylbutyrate	OP	C_20_H_20_O_6_	356.126	356.1282
43	65,531	**Quercetin 4’-*O*-glucoside**	F	C_21_H_20_O_12_	464.0955	464.0924
44	67,531	Sesaminol	L	C_20_H_18_O_7_	370.1053	370.1052

**Bold**, match with compounds found in previous batch, (†), found in olive trees and their products, i.e., olive oil and olives, and (*) for the first time in propolis [12]. **p-HPEA-EA**, p-HPEA-Elenolic acid mono-Aldehyde; Ligstroside-aglycone mono-aldehyde; (Ligstroside-aglycone major form). Class (F: Flavonoids; PA: Phenolic acids; L: Lignans; OP: Other polyphenols; S: Stilbenes).

**Table 3:** Volatile compounds of the ethanolic extract of Spanish propolis (EESP) identified by GC-MS.

Compounds Identified by GC-MS		
RT (min)	Compound	Math Result
8.605	**Benzyl Alcohol**	938
9.988	**Phenylethyl Alcohol**	948
11.172	**Benzoic acid**	925
13.693	**Vanillin** †	929
27.699	4H-1-Benzopyran-4-one, 5-hydroxy-7-metho	913

**Bold**, match with compounds found in previous batch, (†), found in olive trees and their products, i.e., olive oil and olives.

## Data Availability

The data presented in this study are available in this article.
